# Supplementary material for: Vitamin D Modified DSS-Induced Colitis in Mice via STING Signaling Pathway
Source: Biology (Basel). 2025 Jun 18;14(6):715. doi: 10.3390/biology14060715 (PMC12190092; doi:10.3390/biology14060715)
Supplement: Supplementary file 1 [file biology-14-00715-s001.zip › Supplementary Materials S1: 16s-report of gut microbiota/Groups/diff_analysis/TukeyHSD/Tukey_METACYC_rotate.pdf]

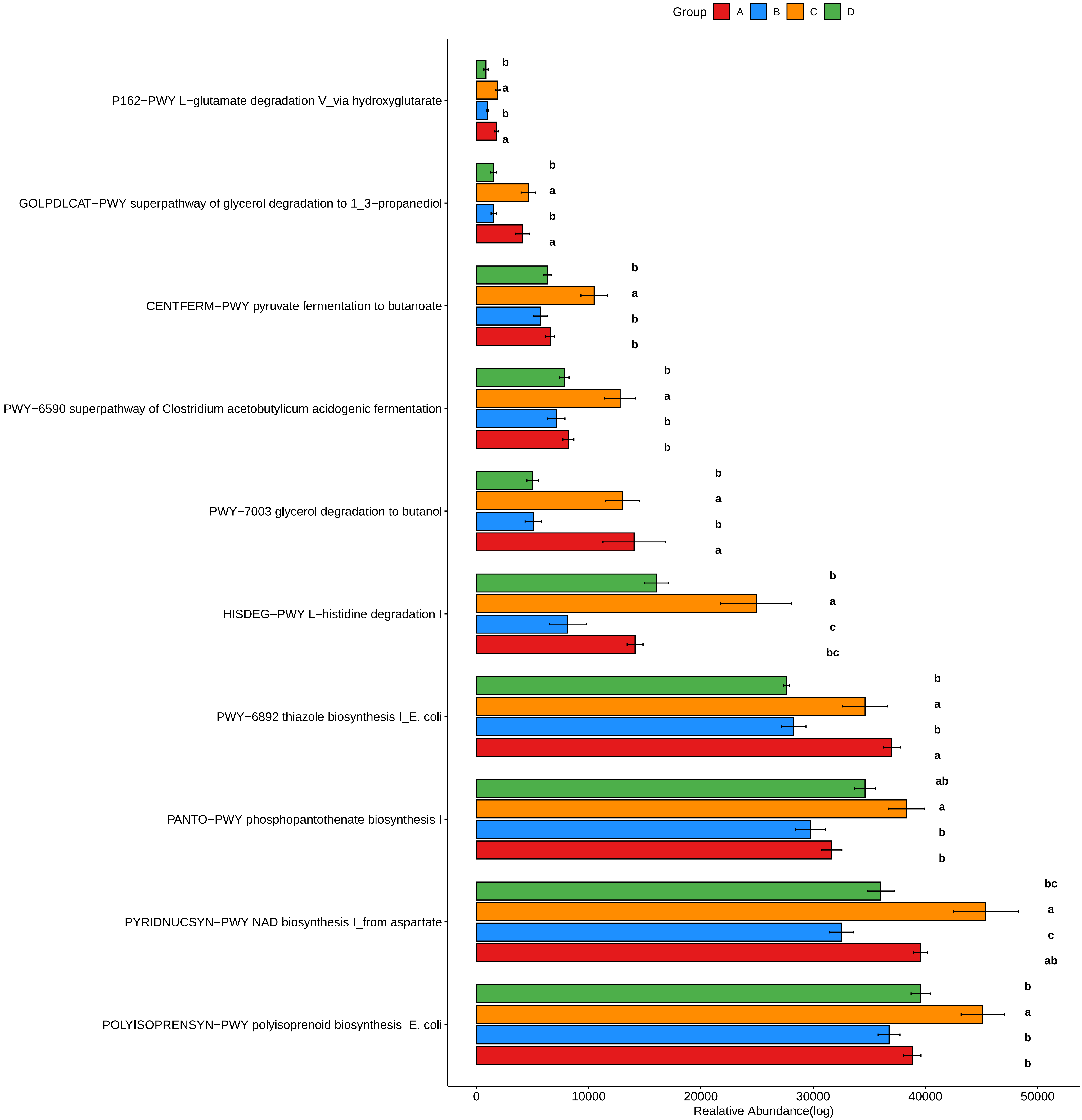

Group
A
B
C
D
P162−PWY L−glutamate degradation V_via hydroxyglutarate
GOLPDLCAT−PWY superpathway of glycerol degradation to 1_3−propanediol
CENTFERM−PWY pyruvate fermentation to butanoate
PWY−6590 superpathway of Clostridium acetobutylicum acidogenic fermentation
PWY−7003 glycerol degradation to butanol
HISDEG−PWY L−histidine degradation I
PWY−6892 thiazole biosynthesis I_E. coli
PANTO−PWY phosphopantothenate biosynthesis I
PYRIDNUCSYN−PWY NAD biosynthesis I_from aspartate
POLYISOPRENSYN−PWY polyisoprenoid biosynthesis_E. coli
b
a
b
a
b
a
b
a
b
a
b
b
b
a
b
b
b
a
b
a
b
a
c
bc
b
a
b
a
ab
a
b
b
bc
a
c
ab
b
a
b
b
0
10000
20000
30000
40000
50000
Realative Abundance(log)
